# From cultured to uncultured genome sequences: metagenomics and modeling microbial ecosystems

**DOI:** 10.1007/s00018-015-2004-1

**Published:** 2015-08-09

**Authors:** Daniel R. Garza, Bas E. Dutilh

**Affiliations:** 1 Centre for Molecular and Biomolecular Informatics, Radboud Institute for Molecular Life Sciences, Radboud University Medical Centre, Geert Grooteplein 28, 6525 GA Nijmegen, The Netherlands; 2 Theoretical Biology and Bioinformatics, Utrecht University, Padualaan 8, 3584 CH Utrecht, The Netherlands; 3 Department of Marine Biology, Institute of Biology, Federal University of Rio de Janeiro, Rio de Janeiro, Brazil

**Keywords:** Biological dark matter, Pan-genomics, data recycling, Eco-systems biology, Metagenome-wide modeling, Virus-host association

## Abstract

Microorganisms and the viruses that infect them are the most numerous biological entities on Earth and enclose its greatest biodiversity and genetic reservoir. With strength in their numbers, these microscopic organisms are major players in the cycles of energy and matter that sustain all life. Scientists have only scratched the surface of this vast microbial world through culture-dependent methods. Recent developments in generating metagenomes, large random samples of nucleic acid sequences isolated directly from the environment, are providing comprehensive portraits of the composition, structure, and functioning of microbial communities. Moreover, advances in metagenomic analysis have created the possibility of obtaining complete or nearly complete genome sequences from uncultured microorganisms, providing important means to study their biology, ecology, and evolution. Here we review some of the recent developments in the field of metagenomics, focusing on the discovery of genetic novelty and on methods for obtaining uncultured genome sequences, including through the recycling of previously published datasets. Moreover we discuss how metagenomics has become a core scientific tool to characterize eco-evolutionary patterns of microbial ecosystems, thus allowing us to simultaneously discover new microbes and study their natural communities. We conclude by discussing general guidelines and challenges for modeling the interactions between uncultured microorganisms and viruses based on the information contained in their genome sequences. These models will significantly advance our understanding of the functioning of microbial ecosystems and the roles of microbes in the environment.

## Introduction

**Fig. 1:**
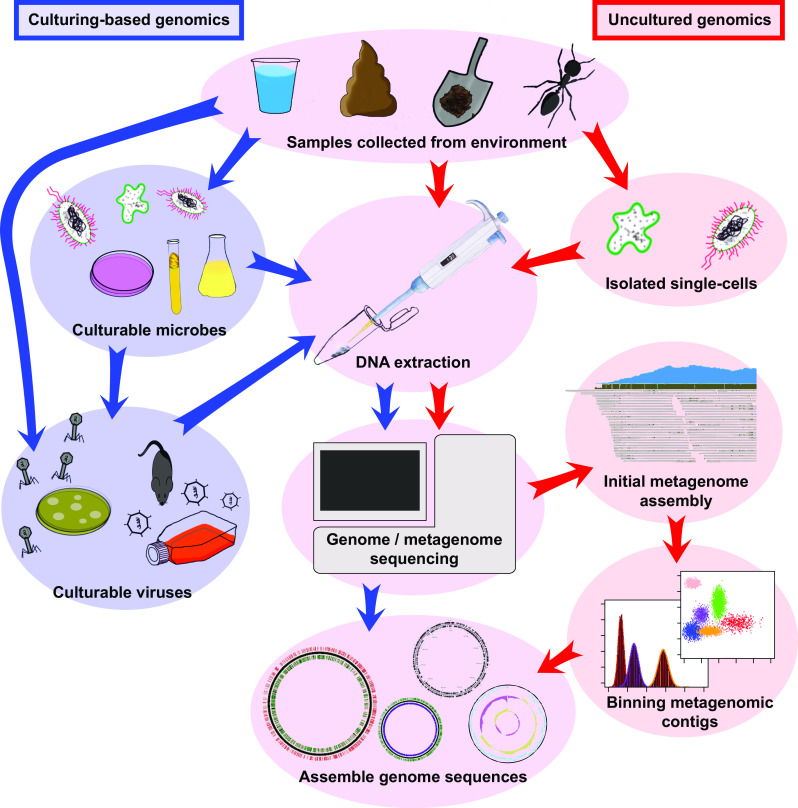
Illustration of simplified pipelines to obtain genome sequences from cultured and uncultured microbes and viruses. There are many variations of each protocol and additional steps, such as filtering samples according to molecular size cutoffs and normalization of data which are not illustrated in this diagram. The purpose is to illustrate simplified general steps to obtain uncultured genomes, which are common in most of the studies discussed in this review

Metagenomics is the study of genetic material recovered directly from environmental samples in an untargeted (shotgun) way. Current developments increasing the depth and breadth of metagenomic shotgun sequencing have facilitated the identification of complete or nearly complete microbial and viral genome sequences from environmental samples without the need to first cultivate these organisms. Here we name these sequences the “uncultured genome sequences” that can either be obtained from metagenomic datasets or from single-cell sequencing. While they frequently have a draft status, and depending on the approach may represent a locally occurring metapopulation rather than a single clone, uncultured genome sequences can supplement the genome sequences obtained by sequencing pure or nearly pure cultures of microbial isolates (Fig. 1), therewith greatly increasing the amount of data that is available for comparative genome analyses. Moreover, by providing reference sequences for the alignment of both known and unknown metagenomic shotgun sequencing reads [1], they greatly enhance the breadth of our understanding of microbial ecosystems. Uncultured organisms may, or may not have close cultured relatives, but isolating complete or nearly complete genome sequences from metagenomes invariably identifies genetic novelty, revealing flexible pan-genomes, genetic variants, and new subpopulations of microbes.

The goal of this review is to introduce some of the recent landmarks of metagenomics in providing new insights into the uncultured microbial biosphere, and highlight the promises and challenges these new genome sequences bring for modeling natural microbial ecosystems. A historic perspective of the discovery of new microbes and viruses before and after metagenomics is given, followed by a discussion of the innovative tools that have been recently used by several research groups to obtain uncultured genomes from metagenomic datasets. Metagenomics is primarily a science of microbial communities, and a key interest is to describe and predict the interactions between different populations of microbes and viruses [2]. Thus, in the further sections of this review we focus on the use of metagenomics and uncultured genome sequences to understand the ecological and evolutionary dynamics of microbial populations within the context of their natural environments. We conclude by discussing the recent developments and perspectives of genome-guided systems biology modeling frameworks to functionally couple the biological knowledge obtained from uncultured genome sequences with systems-level predictions of the dynamics of microbial communities.

## Before metagenomics: culturing-dependent discovery of new microbes and viruses

The first accounts of the microscopic world beyond the resolution of the human eye were made by direct observations of microbes in environments such as water, soil, or diseased tissues. Antonj van Leeuwenhoek, a Dutch tradesman, was the first to build microscopes capable of viewing single-celled organisms. In the late seventeenth century when he reported his observations of “little animals” in water, he was ridiculed by the scientific establishment. Only after his observations were validated by an independent committee did scientists begin to believe that invisible single-celled organisms could be hidden in many habitats in our planet [3]. Before long, microorganisms were recognized as the causative agents of many poorly understood phenomena, particularly in human disease. More powerful microscopes and staining methods were further developed, including the Gram stain in the 19th century, which is still used widely as a first classification scheme for bacteria [4].

Despite the dominance of direct observation and culture-independent methods in early years [5–7], microbiology soon became a science of microbial isolates. After Robert Koch pioneered methods for the isolation of microbial colonies and established postulates to link diseases with causative microbial agents, isolation and cultivation became the most common approach for microbial characterization [8]. Today, many taxonomic and strain typing schemes depend on culturing, as do most laboratory methods for determining the identity and biological characteristics of microbial species.

Virology has followed a path that is very similar to bacterial microbiology. Much of the known viral biodiversity encompasses medically relevant viruses. Before the advances of PCR and DNA sequencing methods, sampling from diseased phenotypes and inoculating into tissue cultures or susceptible animals was the main source of isolation and discovery of new viruses [9]. Additionally, many bacterial viruses (known as bacteriophages) were discovered in rapidly growing, cultivable bacteria, thereby attributing the majority of the recognized bacteriophage biodiversity to fast growing hosts [10]. Thus, by the use of cultivation as a dominant technique in both bacterial and viral microbiology, much of the scientific knowledge has been based on cultivable species, biasing our understanding of microbial biodiversity towards the biology and ecology of the “easy growers” [11].

### Caveats in studying cultured isolates

The study of cultured isolates has propelled microbiological research. The success of culturing microbial species and studying them in isolation is a consequence of the difficulties that would be involved in analyzing them within their natural environment, which is complex and contains many unknown variables. Reproducibility of results, control of external variables, and simple design of laboratory experiments are all advantageous properties that are greatly facilitated in pure culture studies. Nevertheless, studies of environmental microbes and viruses repeatedly confirm that the large majority has not been cultured and is thus poorly understood. The early studies that pointed to an abundance of unculturable microorganisms in the environment were largely forgotten by the scientific community [12–14]. As a result, the development of modern culture-free methods including metagenomics, have sometimes led to surprises in the past 20 years. For example, by visualizing and counting the microscopic biological particles in the environment and comparing these counts to the number of archaeal and bacterial isolates, or to the number of phage plaques that grew on a bacterial lawn, a great numeric discrepancy was observed between what was counted in the wild, and what could be cultured on a plate [10, 11]. Different environments, such as seawater, soil, or marine sediments, showed that only about 0.01–1 % of the microorganisms seen in the microscope could be isolated on artificial media, while the vast majority remained intractable to culture-dependent techniques. These discrepancies have been named the “great plate count anomaly” [11] and the “great plaque count anomaly” [10], respectively. Clearly, we do not yet truly understand microbial biodiversity, which begs basic questions such as, which bacteria or viruses are out there? What is a microbial species? How do microbes and viruses interact with each other? And how do they interact with their environment?

It is particularly relevant to broaden the phylogenetic breadth of cultured isolates in order to have more diversity available for experimental testing [15]. Moreover, since the majority of viruses in natural environments consist of bacteriophages, having a greater diversity of cultured bacterial isolates will also allow for a higher throughput in virus isolation strategies [16]. Given the observations of a vast, uncultured majority of microbes and viruses as outlined above (the great plate/plaque count anomaly), a natural question to ask is “Why do most bacteria, archaea, and viruses not grow in synthetic media?” [17]. Another related question is “How can we increase the recovery of environmental microbes in pure culture?” Many authors who discuss these and similar questions suggest that there are no single answers, and that many answers are applicable only to specific taxonomic groups or hold only in particular environments [17]. Among the commonly suggested causes for the plate count anomaly, we can list (1) lack of essential nutrients in the isolation media [18–20]; (2) lack of an essential biological interdependency with other species, such as auxotrophs or obligate mutualists [21–23]; (3) poor correlation between the in vitro growth condition and the environment: e.g. the media are too rich or too poor in nutrients, or they have inappropriate pH, salinity, or temperature [19, 24, 25]; (4) microbe-specific features, such as small non-cultivable cells, or extremely slow growers [26]. Some of these causes are interrelated and may be addressed together (see below).

### Methods to increase the plate count

Early approaches to increase the plate count were based on extensive testing of different media, such as the R2A media for drinking water biofilms [24], and low-throughput screening for compounds and co-factors that could increase the plate count for different environments [27]. Promising technologies are being developed, some of which can be extended to high-throughput approaches [28, 29]. These technologies allow for many different conditions and samples to be screened in parallel. Simultaneously screening bacterial phenotypes in different conditions is one example of a high-throughput approach that can be used to identify optimal culturing conditions [30]. Other approaches involve the cultivation of bacteria in their natural environment or the use of supplements and specific growth factors such as iron-chelating siderophores [19, 20]. Fe(II) is severely limited in most aerobic environments and some bacteria release siderophores to scavenge for Fe(II), which is then transported back into the cells. Siderophores from neighboring species induce growth of uncultured marine bacteria. By inoculating marine bacteria with high concentrations of Fe(II) as a surrogate for siderophores, D’Onofrio et al. [20] reported the isolation of many colonies of previously uncultured bacteria, including three with 16S rRNA gene sequences that were highly divergent from any known species [20].

Allowing small metabolites or signaling molecules from the natural sites of isolates to diffuse into inoculated surfaces was shown to recover up to 50 % of bacteria from some environmental samples, where traditional methods would only recover 0.01–0.05 % [18, 19]. In order to achieve these expressively higher colony yields, diffusion chambers built with washers, sandwiched between 0.03 µm pore membranes were used, and incubated together with the sediment collected from marine environments in a marine aquarium. Some bacteria grow in diffusion chambers only when paired with so-called “helper” species [22]. One of these bacteria, *Psychrobacter* sp. strain MSC33, started growing in isolation after successive co-cultures with its helper strain, *Cellulophaga lytica*. After acquiring the capacity to grow in isolation, *Psychrobacter* MSC33 in turn could be used as a helper strain for other bacteria. This phenomenon was reproduced with other strains that could only grow in co-culture and, importantly, it was also observed in rich media, suggesting that nutrient limitation was not the underlying mechanism for the initial inability of these strains to grow in isolation. Indeed, the authors identified a five-amino-acid signaling peptide, LQPEV, as responsible for inducing the growth of the otherwise unculturable *Psychrobacter* [22].

One example of nutrient interdependency as the limiting factor for obtaining pure bacterial cultures was found with *Treponema primitia*. This bacterium is a hydrogen consuming, carbon dioxide-reducing homoacetogenic spirochete from the termite hindgut, and relevant for the host due to nitrogen-fixing and acetate production functions. Graber and Breznak [21] showed that *T. primitia* only grows when folate is available and they suggest that this nutrient is provided by other microbial members in the termite hindgut [21].

A promising device for high-throughput isolation of microbes from natural environments is the iChip, which consists of hundreds of miniaturized diffusion chambers [29]. Recently a previously uncultured proteobacterium, *Eleftheria terrae*, was discovered by using this technology [25]. This bacterium produces a potent antibiotic named Teixobactin, which has was found to be active against Gram-positive bacteria not amenable to treatment, and is being suggested as an effective drug against methicillin-resistant *Staphylococcus aureus* MRSA [25].

### Genome-guided culturing efforts

Finding the right culturing conditions or hosts to isolate novel microbes and viruses can be guided by mining uncultured genome sequences for clues of potential nutrient requirements. An example is provided by the SAR11 clade, which is the most abundant clade of heterotrophic bacteria in the ocean. As of 2002, these bacteria were known solely from evidence based on environmental sequencing data [31]. Although SAR11 isolates were obtained by using sterile seawater with several supplements [32], genome mining showed that these bacteria lacked assimilatory sulfate reduction genes, thus requiring exogenous sources of reduced sulfur, such as methionine or 3-dimethylsulphoniopropionate (DMSP) for growth. DMSP is provided by other plankton members and its addition to the culture media significantly increased the biomass yield of SAR11 bacteria [23]. These results suggest that the availability of complete or nearly genome sequences for different representatives of the uncultured groups could guide isolation strategies for these different microbes.

**Fig. 2:**
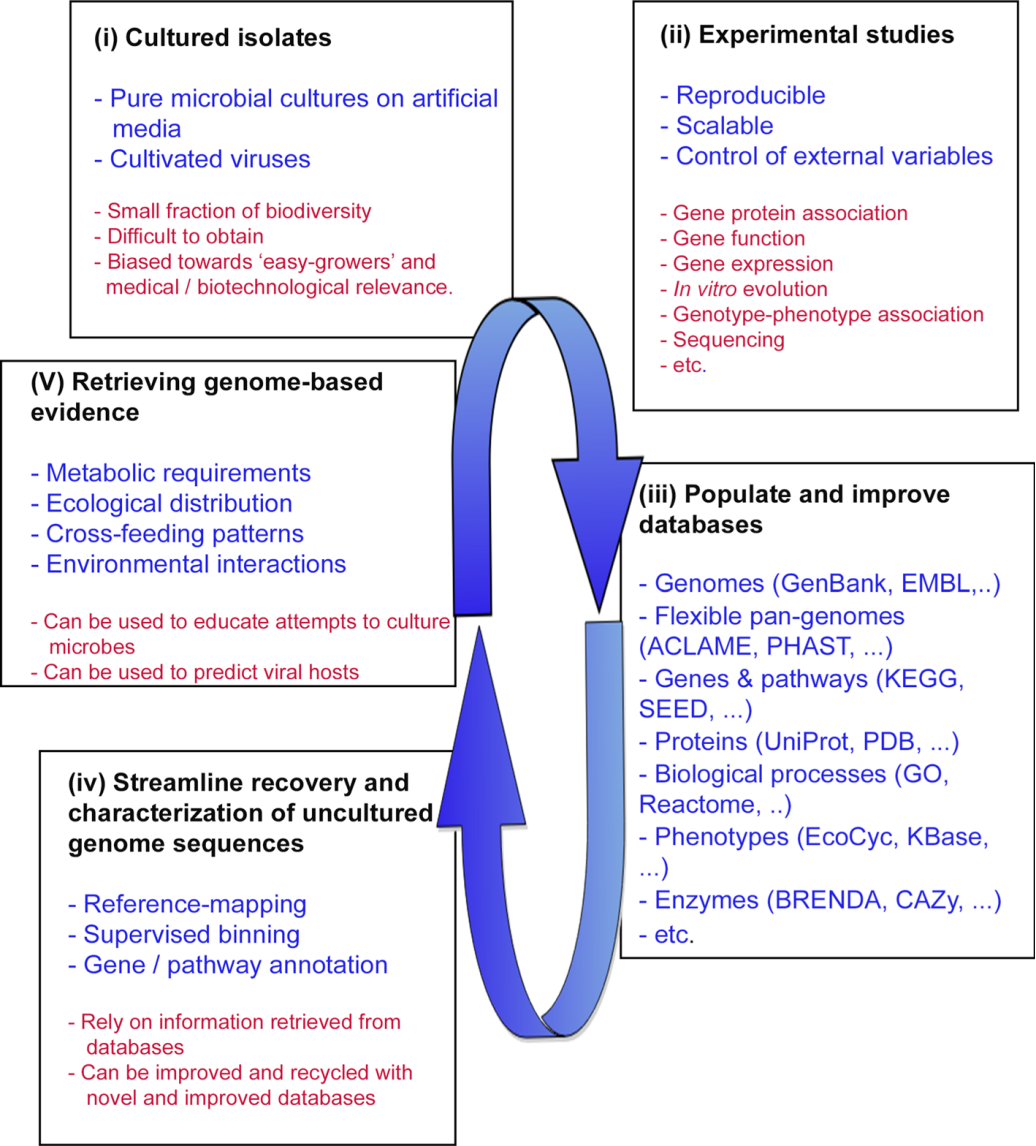
Diagram of the feedback loop between experimental studies on cultured isolates and genome-based evidence retrieved from sequenced genomes. Uncultured genomes can educate genome-guided culturing attempts, which are suggested in the main text

Besides providing access to uncultured genome sequences, metagenomics can also be used to study microbes and viruses in the context of their interactions with other members of the biological community. This makes metagenomics a fundamental tool to be integrated with environmental microbiology and the study and discovery of novel microbial biodiversity. Ideally, there is a feedback loop between bioinformatic approaches that obtain uncultured genome sequences from shotgun metagenomic datasets, and the laboratory where these genome sequences are exploited to guide the cultivation efforts of new microbial species (Fig. 2). First, the phenotypic and genetic characterization of cultured microbial isolates can populate databases with data that help to increase the accuracy of the information that can be obtained from their genome sequences. Second, obtaining uncultured genome sequences from metagenomes can uncover the gene composition of a species and its putative phenotype space, providing meaningful information for attempts to isolate microbial species. Moreover, the distribution across environments can also be retrieved from metagenomic analyses, which can be used to predict ecological interactions and lifestyles.

Genome-guided culturing is a vastly underexplored area in the field of metagenomics. Examples of uncultured genomes that could be amenable to these approaches include the candidate phyla OD1, OP11, and BD1-5 [33]. These three candidate phyla are part of a monophyletic group of widespread uncultured bacteria that have only recently been recognized by metagenomic sequencing, and were shown to comprise a super-phylum that encompasses an estimated 15 % of the bacterial domain [34]. Genomic evidence suggests that these bacteria have small genomes and may depend on other community members for essential nutrients [34, 35]. Deep sequencing revealed that besides remarkably small genomes, they lack many known biosynthetic pathways [36] and analysis of their ultrastructure suggests that they are indeed naturally ultra-small cells with median volumes of 0.009 µm^3^, but are biologically active [35]. Enrichment for a member of the BD1-5 bacteria in a chemostat containing a mixed culture [37] suggests that these bacteria could be amenable to cultivation under laboratory conditions. Even before uncultured genome sequences were available, Harris et al. [33] suggested using the environmental distribution patterns inferred from 16S rRNA amplicon sequencing to develop isolation strategies for these groups.

To conclude, cultured isolates are critical for reproducible experimental studies. Isolates are useful for many biotechnology and health applications, such as genotype-phenotype screening, gene knockouts, screening for secondary metabolites, and phage-host assays. Nevertheless, there are many difficulties in the process of obtaining cultured representatives for the vast diversity of microorganisms and viruses, which can thus only be studied by using culture-independent methods.

## Metagenomics approaches to study new microbes and viruses

### Marker genes and the phylogenetic identity of uncultured bacteria and archaea

Estimates of the size of the environmental microbial and viral biodiversity that remains to be discovered are vast. In bacteriophages, for instance, it has been estimated that there are on the order of 100 million undiscovered types with possibly billions of new genes [38]. Knowledge of the microbial world is dependent on tools that increase the signal-to-noise ratio of the uncultured genome sequences in metagenomes that represent the hidden members of microbial communities. While the first studies that addressed uncultured microorganisms could only infer their presence by the shapes and stains under the microscope, in the past 50 years, developments in molecular biology have provided advanced tools to survey and quantify this hidden majority. The developments of the polymerase chain reaction (PCR), fluorescence in situ hybridization (FISH) [39], advances in DNA sequencing technology, and use of the 16S rRNA gene as a taxonomic marker [40, 41], have enabled the genetic identification of bacteria and archaea that are found in different environmental samples. By isolating DNA samples from whole communities of microorganisms and further amplifying and sequencing fragments of the 16S rRNA gene selected with degenerate primers, the genetic identity of a representative portion of the microbial community can now be known (for a review see ref [42]).

The 16S rRNA gene and other taxonomic marker genes have provided the means both to identify microbes by sequence similarity, and to cluster them into taxonomic groups in a phylogenetic context. Moreover, these marker genes have enabled estimates of the proportion of biodiversity that remain uncultured, revealing whole phyla that lack cultured representatives [34, 43]. Importantly, these phyla cannot be classified with conventional taxonomic approaches, which rely on polyphasic phenotypic and genetic typing schemes that are currently inaccessible for uncultured microbes [44, 45]. Uncultured groups suggested by this method are thus termed candidate phyla. Currently, more than half of the known bacterial and archaeal phyla lack cultured representatives.

### Uncultured genome sequences come into play

A metagenome consists of the genomic sequences of all the organisms present in a given environment. Metagenomics can be defined as the application of high-throughput sequencing and analysis pipelines to elucidate a representative, random fraction of the genome sequences in a biological sample [46].

Before shotgun metagenomics, environmental sequencing efforts focused on the processing of amplified phylogenetic marker gene sequences. Since then, metagenomics has evolved into the application of shotgun sequencing aimed at obtaining sequencing reads from a comprehensive fraction of the nucleic acids in a sample (for general reviews about metagenomics see refs. [47–49]). Some of the first metagenomic studies consisted of shearing environmental DNA from soil samples into large fragments, cloning these fragments into BAC vectors and screening for functional traits [50, 51]. This approach of enriching and screening for functional genes is now named functional metagenomics to differentiate it from approaches that were aimed primarily at discovering the global sequence content of environmental samples [52–54].

One of the first comprehensive shotgun metagenomics studies was conducted on eight large water samples from different sites of the Sargasso Sea [54]. Fosmid libraries were generated from isolated and fragmented DNA from this community and sequenced by the dideoxy chain-termination method (Sanger sequencing). More than 1.5 Gbp of sequences were generated, many of which could be assembled into scaffolds, suggesting the presence of countable, discrete species rather than a genomic continuum [54]. These were among the first sequences of uncultured microorganisms and contained partial genome sequences from phyla that had no cultured representatives, such as the SAR86 clade. Using the term “genomic species”, the authors clustered genome fragments by using a similarity cutoff and found direct evidence that at least 451 different uncultured genome sequences were sampled. Additionally, many new genes were discovered and assigned to functional categories.

Since these first endeavors, DNA sequencing of microbial communities has evolved from the Sanger sequencing methods, which rely on a labor-intensive cloning process, to Next Generation Sequencing (NGS) technologies such as the 454/Roche, Illumina/Solexa, and Ion Torrent/Ion Proton platforms [55]. These short read approaches are particularly suited for taxonomic and functional profiling of metagenomic samples, as they provide a random sample of the sequences therein [56, 57]. Thus, and as a result of the rapidly decreasing cost of short read sequencing, such profiling analyses have been the driver of the field of metagenomics in the past decade. With the further decrease in cost and increase in sequencing volumes and read lengths, for example by PacBio and Oxford Nanopore sequencing technologies, assembly of (draft) uncultured genome sequences is now becoming increasingly accessible. We will discuss new promising methods for identifying and characterizing these uncultured genome sequences in the paragraphs below.

### Bioinformatic approaches to obtain uncultured genome sequences

Assembly of uncultured genome sequences from complex shotgun metagenomes is progressing with the rapid development of new sequencing methods and bioinformatics pipelines [58]. Below we will review approaches that have been developed and used by several research groups to build uncultured genome sequences de novo. A metagenomic sample consists of random fragments of multiple genomes from different organisms. These genomes contain signals such as phylogenetic or sequence based signals that have been acquired in the course of evolution [59–61], signals that are the result of the ecological process [62, 63], or signals resulting from the sampling strategy [64]. These signals may be exploited to group metagenomic sequence fragments belonging to the same organism together, in order to bin and assemble the original uncultured genome sequences.

The naturally occurring sequence diversity of microbial genomes, whether derived from co-existing strains or from a (viral) quasispecies, often prohibits the assembly of longer contigs [65]. In bioinformatics, the process of grouping genomic fragments such as reads or assembled contigs putatively derived from the same organism based on sequence signals, is called binning, and many bioinformatic tools are available to do this [64, 66–69]. From a bioinformatics point of view, the most important signals available for contig binning are: homology to a reference sequence, paired sequencing read information, oligonucleotide composition, and differential abundance patterns across metagenomic samples. Moreover, an experimental approach that was recently developed exploits Hi-C, a technology that was developed to detect chromosomal organization in eukaryotic cells, to identify DNA sequences that are co-localized within microbial cells of an environmental sample [70–72]. We expect that additional experimental and bioinformatic approaches will be developed for binning uncultured genome sequences from metagenomes, as the opportunities for interpreting and analyzing uncultured genome sequences improve (see below).

Binning approaches can be classified into supervised and unsupervised methods. Supervised methods generally use a reference database of known genomes as a training set, and apply statistical classification methods, such as hidden Markov models [73, 74] or similarity/distance matrix models [75], to classify reads. These classification approaches can be used to remove or isolate clusters of sequence fragments according to a specific signal and thus reduce the complexity and size of the assembly challenge. Supervised methods can also be used to classify reads and assemble genomes from the resulting bins [73, 76, 77].

Homology-based signals consist of aligning sequencing reads or contigs to a reference sequence that can consist of the genome of a known species, or of contigs assembled from the same or a similar metagenome [78–80]. An obvious limitation of the supervised approaches is that they are restricted to discovering genomes that are similar to the genomes which were used as training sets, making them unsuitable to discover completely novel genome sequences. Nevertheless, these algorithms tend to improve as an increasing number of reference sequences become available, particularly of uncultured organisms, and they can be continuously calibrated towards more adequate training sets.

Unsupervised methods do not depend on a database of known reference genomes [81]. These methods are generally dependent on sequencing strategy, or on sequence content and sample composition. For example, as in cultured genome assembly, paired sequencing reads are commonly exploited for scaffolding assembled contigs, by mapping read pair sequences to the assembled contigs, and linking the contigs that share many paired sequencing reads [82]. The computational performance of alignment-based approaches that depend on alignment of many sequences is also rapidly improving thanks to innovative bioinformatic tools [83, 84].

Binning signals based on sequence content include the percentage of G and C nucleotides in the contig, as well as the oligonucleotide usage profile that are both relatively consistent along the length of the genome. For these approaches, larger fragments or contigs result in better approximations of the genomic oligonucleotide usage profile, and better binning. These alignment-free methods can be very fast and memory-efficient, because binning is achieved by simple binary vector operations, which computers perform extremely fast. An example of an unsupervised approach that exploits oligonucleotide usage profiles is emergent self-organizing maps (ESOMs) [85–88].

Signals that are based on differential abundance patterns of a genome within or across metagenomic samples exploit the consistency in the expected depth of coverage of contigs that are derived from the same genome. Because different genomes are present in different frequencies in a sample, fragments from one genome are expected to have the same depth of coverage in the metagenomic dataset, thus reflecting the abundance of that genome in the original sample. If multiple metagenomic samples are obtained from a similar environment, each with variations in the abundances of the different members of the microbial community, the depth of coverage of contigs derived from one genome is expected to vary consistently across samples. This allows for fragment binning based on co-abundance across multiple metagenomic samples [64]. This differential abundance signal, in combination with oligonucleotide usage profiles were used to identify 49 nearly complete uncultured bacterial genome sequences from an acetate-amended aquifer [62].

The assembly of high quality uncultured genome sequences from metagenomic datasets is still a relatively low throughput process that usually yields only a few nearly complete genomes. In part, this depends on the sequencing volume and the species richness of the sampled community, which together determine the expected assembly depth of the uncultured genome sequences. The greatest bottleneck, however, is the effort that goes into finishing a genome sequence. For bacteria and archaea, the completeness and redundancy of an assembled uncultured genome sequence consisting of a cluster of binned contigs, can be assessed by identifying universal single copy marker genes [34, 89, 90]. The percentage of these universal genes identified in the assembled genome corresponds to the expected genome completeness, while duplicates among these single copy genes indicate redundancy. For viruses, such universal marker genes are not available, and currently the most reliable way to establish completeness of an assembled genome sequence is by validating that the assembled contig represents a circular genome [63, 91, 92]. However, with new bioinformatic advances [64, 66–69], the recovery of uncultured genome sequences, whether in draft or complete form, is increasingly yielding new knowledge about natural microbes and viruses, as outlined below.

## Examples of landmark uncultured genome sequence assemblies

Tyson et al. [53] were the first to assemble nearly complete uncultured genome sequences from a metagenome library of small-insert plasmid clones. The isolation and reconstruction of genomes was possible because the sampled community consisted of low-complexity biofilms containing few different species. After an initial assembly of shotgun reads, the larger contigs were binned based on the GC content and read coverage, allowing the recovery of nearly complete genomes of *Ferroplasma* type II and *Leptospirillum* group III. These organisms had never been cultivated. With these genome sequences, the phylogenetic origin of these organisms could be inferred, as well as their relative dominance across similar samples. Based on gene annotation, the authors suggested metabolic functions across genomes and inferred ecological cross-feeding interactions between organisms involved in the community’s carbon and nitrogen cycles [53].

Narasingarao et al. [93] obtained scaffolds from Sanger sequencing of size-fractionated samples from a hypersaline lake in Victoria, Australia. By binning these scaffolds based on the GC content, they found two distinctive GC profiles from very small cells that passed a 0.8 µm filter but were retained at 0.1 µm pore sizes. Using a phylogenetic binning approach, they recovered two draft uncultured genome sequences, which were representatives of a totally new branch of uncultured *Halobacteria*. Nearly 60 % of the predicted genes in these archaea had no homology with proteins in Genbank and they exhibited a very distinctive codon usage profile when compared to other archaea [93]. Although most genes in these microorganisms were unknown, the fraction of annotated genes suggested a predominantly aerobic heterotrophic lifestyle and also the presence of a complete pentose phosphate pathway, which had not previously been found in archaea [94]. These genomes were compared with other databases, suggesting that these archaea belong to a new, widespread class for which the authors coined the name “*Nanohaloarchaea*”. At least eight distinct clades of this class have been found in hypersaline environments across different continents [93].

In a recent article, Spang et al. [89] reconstructed three partial uncultured archaeal genome sequences from marine sediment metagenomes, which together comprise the *Lokiarchaeota*, a candidate archaeal phylum that, based on phylogenomic analyses, encompasses the base of all eukaroytes. Comparative genomic analyses of the uncultured genome sequences identified eukaryotic signature genes, including genes that are involved in membrane remodeling and vesicular trafficking. Based on these genomic observations, the authors proposed that the uncultured *Lokiarchaeota* contain a complex cellular machinery that may have facilitated the acquisition of the proto-mitochondrial endosymbiont into the ancestor of all eukaryotes. This example from the field of evolutionary biology highlights that metagenomic discovery of uncultured genome sequences can impact all areas of biology and is not limited to microbial ecology.

The new taxonomic groups identified by metagenomics can be vast. In a recent study, Brown et al. [34] assembled 8 complete and 789 draft genome sequences from tiny uncultured bacteria in size fractioned samples from an aquifer adjacent to the Colorado River. These genomes are members of a new super-phylum of at least 35 different bacterial phyla that was estimated to encompass 15 % of the bacterial domain [34]. Phylogenetic evidence suggests that this phylum forms a monophyletic group, which the authors named the candidate phyla radiation (CPR). Analysis of these uncultured genomes revealed many unusual features. For example, several nearly universal ribosomal genes [95] were absent from many draft genomes, such as rpL9 that was not detected in any of the 16 uncultured genome sequences from the WS6 candidate phylum [34]. Although the uncultured genome sequences were estimated to be only ≥50 % complete (median completeness 91 % for WS6), the authors suggest that it is highly unlikely that all draft genome sequences lack the same gene by chance. Moreover, analysis of the 16S sequences of these uncultured genomes revealed the wide-spread presence of large introns within the 16S rRNA genes. It was suggested that the commonly used primers for 16S amplicon sequencing would miss a large fraction of these bacteria due to primer mismatching and the presence of these introns [34].

It may be expected that similar metagenomic investigations into the vast, uncultured microbial biosphere, including archaea and viruses that remain poorly represented in current databases, will yield many new and exciting discoveries in the near future.

### Minority groups

It is important to realize that the uncultured genome sequences obtained from metagenomes represent consensus sequences of closely related genomes [65]. If there are multiple highly similar strains within a sample, metagenome assembly approaches tend to collapse these genotypes into a single consensus sequence. Indeed, most genome sequences that are available today represent consensus genome sequences, including the reference genomes of many bacteria and animals. For most applications this is sufficient and allows firm conclusions to be drawn. However, some applications may require genotypes of individuals, for example in population genomics, and an alternative to obtain uncultured genome sequences of such individual genotypes is to perform single-cell sequencing [15, 96, 97]. In this approach, single-cells are separated by cell sorting, their genomic content is randomly amplified by multiple displacement amplification (MDA) that exploits the phage-derived Φ29 DNA polymerase and random short primers, and subsequently sequenced. Several groups have used single-cell sequencing combined with metagenomics to simultaneously obtain consensus sequences and individual genotypes [98–100]. Like with genome assembly from metagenomes, the completeness of single cell genomes can vary widely from <10 to 98 % [15, 96–98, 100].

The identification of minority groups that are under-represented in the community, and thus in the bulk of shotgun metagenomic sequencing reads is a challenge when identifying uncultured genome sequences in metagenomes. Single cell sequencing may not be a good approach in these cases because isolation strategies tend to favor the majority, although the identity of cells can be determined by using probes before sequencing [101–103]. The genome sequences of some minority members from a marine community were recovered by using mate-paired reads sequenced on a SOLiD platform [82]. In this study, 58.5 Gb of mate-paired reads were generated and assembled into contigs. The mate-pairing information was used to link the contigs into interconnected graphs, and oligonucleotide usage profiles and read-coverage statistics were used to bin the contigs into larger linear scaffolds. Several candidate genomes were assembled with this approach, including a member of uncultured group II *Euryarchaeota*, whose genome indicated that this microbe is photoheterotrophic with aerobic metabolism and the ability to degrade lipids and proteins [82]. Other uncultured genomes of this group were later sequenced and assembled from metagenomic fosmid clones, confirming similar features [104]. This approach of deep mate-paired sequencing combined with partial-assembly and binning based on compositional features was also used to assemble 15 draft genomes from samples enriched for biomass-degrading microbes from cow rumen [99]. The completeness of one of these genome sequences was assessed by single cell sequencing, showing that a significant part of the genome was present in the original draft assembly and that no spurious reads had been incorporated. These results demonstrate the validity of this assembly pipeline to produce draft genomes of minority groups within the microbial community.

A similar approach for obtaining the uncultured genome sequences of rare minority groups uses binning based on the relative depth of coverage of fragments from two different DNA extractions of the same sample [64]. This approach was followed by principal component analysis of tetranucleotide usage profiles, and information from paired-end reads were used to isolate 13 nearly complete genomes, including four rare genomes (0.06–1.58 % relative abundance) of uncultured representatives of the TM7 phylum [64].

### Data recycling

In the examples above, metagenomic datasets were newly sequenced and analyzed to discover species in environments that were of particular interest to the researchers. Due to the invaluable efforts of these and other research groups, many metagenomes are now becoming available in the public databases that can be used in secondary analyses. Public databases [105, 106] now contain thousands of metagenomic datasets that can be mined for novel microbial and viral genome sequences. The opportunity for data recycling is strongly driven by the development of new bioinformatic tools and methods for metagenomic analysis. We turn now to some significant examples of uncultured genome sequences that were obtained from recycled datasets.

Cross-assembly (crAss) of different samples from similar environments is one example of a strategy that can point to co-occurring sequences that are shared between environments and may not be identified with other methods such as reference mapping [67]. Our group cross-assembled previously published viral metagenomes of human fecal samples from four homozygotic female twin pairs and their mothers, and found a previously unknown viral sequence that was highly prevalent in human gut microbiomes from different continents, named crAssphage [63]. Up to 24 % of the viral shotgun metagenomic sequencing reads in samples from Korea, and up to 22 % of the reads in unrelated total fecal community metagenomes from USA aligned to the crAssphage genome sequence. The complete genome assembly and the metagenomic context in which it was isolated allowed the prediction of candidate host species, suggesting that it may infect *Bacteroides* hosts.

An alternative approach to analyze multiple metagenomic datasets was used to extract co-abundance gene groups (CAGs) from 396 gut metagenomes [107]. In this approach, metagenomes were first assembled and genes extracted to create a comprehensive non-redundant gene catalog of almost four million gut microbial genes. Genes were then picked randomly, and the abundance profiles across the 396 gut metagenomes of all other genes was compared to the query gene by using Pearson correlation. Highly correlating genes (*r* > 0.9) were iteratively grouped into CAGs, and their abundance profiles averaged until the CAG stabilized. The size distribution of CAGs showed a bimodal distribution with peaks at approximately 50 and 1700 genes, respectively. The CAGs that contained more than 700 genes were re-assembled, and 238 of those yielded genome sequences that met the criteria for high-quality draft genome sequences as defined by the Human Microbiome Project. A total of 181 of these uncultured genome sequences were derived from species that had no previously sequenced representative. Many of the smaller CAGs, potentially representing bacteriophages and mobile genomic elements such as plasmids or integrons, were observed to be dependent on the large CAGs, i.e. they were only present in the samples if the larger CAG was also present [107].

Metagenomics and omics-related approaches are increasingly advancing fields ranging from human and veterinary medicine, to microbial ecology and evolutionary biology. The availability of data and new analytic approaches not only provides new uncultured genome sequences as discussed above, but also enables the characterization of novel clades of archaea, bacteria, and viruses. Identifying the genome sequence of an uncultured organism allows us to ask questions about its diversity, genomic evolution, preferred environments, relative abundances, and co-occurrence with other species. For example, a recently published web tool, Phage Ecol-Locator, allows the investigation of bacteriophage genes across environments in order to answer questions about phage biology, lifestyle, and ecology [108]. These and other questions can be addressed by leveraging publicly available metagenomic datasets. We expect that new tools for metagenomic data recycling will increasingly become available to exploit the knowledge contained in large public databases, with the potential to describe the identity, evolution, and ecological interactions of cultured, as well as uncultured microbes and viruses.

## Top-down approaches to study uncultured genome sequences

Metagenomes can be studied by using top-down and bottom-up approaches. Top-down approaches are based on metagenome-wide statistical patterns that are obtained from the sequence fragments of metagenomic reads, and can, for example, be used to study the structure of the ecosystem, as well as the identity and relative abundances of microorganisms [109]. Bottom-up approaches begin from flexible pre-defined structures of the system, such as genome-scale metabolic models and aim to mechanistically reconstruct patterns and signals that can be measured from the system as a whole by integrating its constitutive parts into a model [110]. Bottom-up approaches will be discussed in a further section.

Obtaining a metagenomic sample, i.e. a random, minimally biased sample of the genomic sequence content of a microbial community, allows for direct and statistical estimates of ecological and evolutionary variables that help explain the structure and function of the microbial ecosystems [78, 111]. With more and better metagenomic data becoming available from sites across the planet, there is an unparalleled wealth of data available in the digital space for scientists to generate, test, and evaluate new hypotheses about microbial ecosystems [112]. Examples of ecological and evolutionary parameters that can be studied in metagenomic datasets include microbial species abundances, richness, evenness, and diversity [113, 114]. Moreover, eco-evolutionary processes can be studied, including competition, cooperation [115, 116], Red Queen dynamics [117, 118], structure and function of communities, as well as patterns of assembly, colonization, and composition of the microbiota [119–121]. Below we outline some of these patterns and emphasize that metagenomics provides not only a comprehensive window to discover and isolate new uncultured genome sequences as outlined above, but also provides the principal data to characterize the ecological context in which these genomes are found.

### Global abundance and distribution patterns

The ecological context of uncultured organisms can be studied by exploiting metagenomic datasets. Many discoveries in this young field have changed established textbook frameworks of microbial relationships with the earth’s physics and chemistry, revealing a less biased view of the structure and function of microbial ecosystems. Light harvesting in the ocean is one example where non-chlorophyll pathways based on bacteriorhodopsin were shown by metagenomics to be a widespread mechanism in the ocean, not only limited to *Proteobacteria* or *Archaea* [54, 122]. Another example is the elucidation of the biogeography and ecology of specific uncultured microbial groups. For example, a group of archaea, (previously called *Crenarchaeota* because of a somewhat close relationship with this phylum [123, 124] but now known as *Thaumarchaeota*, see below) was found by metagenomics to be present in many different environments, such as freshwater [125], sediments [126], ocean water [54], and the digestive tract of aquatic and terrestrial animals [127, 128]. One representative was cultivable in a marine aquarium when grown as a symbiont to the sponge *Axinella mexicana* [127]. Several genomic surveys and later the cultivation of one marine representative of this phylum showed that many of these species encoded ammonia-oxidizing genes [129, 130]. Given the abundance of this phylum in several environments, they have recently been suggested to be major players in the global cycling of nitrogen through ammonia oxidation [131]. Before this group was discovered, ammonia oxidation was thought to be performed almost exclusively by autotrophic ammonia oxidizing bacteria [132]. Later, the assembly of several uncultured genomes and genomic evidence from different sequencing projects rooting this group further apart from the *Crenarchaeota*, led to the recognition of a new archaeal phylum, the *Thaumarchaeota* [133].

### Niche-driven and neutral community assembly

Metagenomic data can be used to determine the mode of assembly of a microbial community. Processes of assembly are relevant to the study of community ecology because they indicate which forces have shaped biological communities and likely influence their structure and function [134]. Two different types of processes are commonly distinguished that shape the composition of microbial ecosystems: deterministic niche-driven, and stochastic neutral processes [135]. Both processes, and combinations thereof, can predict the distribution curve of the relative abundances of species. If a neutral stochastic process has shaped the community, the relative abundances of species are expected to fit a zero-sum multinomial (ZSM) distribution [136, 137]. In the niche-driven process, species are related to environmental changes and the relative abundances are expected to fit a log-normal or a zipf distribution [138]. In the healthy lung, for example, the composition of the microbiota was shown to fit a neutral model with species derived mainly from the oral cavity, while samples from the lungs of patients with cystic fibrosis and idiopathic interstitial pneumonia could not be explained by the neutral model [139]. Mendes et al. [140] compared soil and soybean rhizosphere microbiomes and found a log-normal distribution in the rhizosphere community, while the bulk soil community fit the ZSM distribution. Metagenomics has provided evidence of niche-driven or neutral-processes in several other environments [141–143].

### Biodiversity and ecosystem stability

Biodiversity is another important ecological parameter that can be measured by top-down metagenomics. Biodiversity can be defined as the species richness, i.e. the number of different species that are present in an environment; as the relative abundances of the different species; or as the evenness, a measure that incorporates the phylogenetic breadth of the species [144]. Biodiversity is often related to the stability of an ecosystem [145]. This is the basis of the insurance hypothesis, in which greater diversity insures ecosystems against losses of functionality due to environmental fluctuations and perturbations [146, 147]. Uncultured bacterial and archaeal genomes can be readily inserted into a biogeographic and evolutionary context by comparing their marker genes across these datasets. Data for species richness in microbial ecosystems based on marker genes provides a wide spectrum of information about their distribution patterns, as well as the alpha and beta diversity, and can shed light on migration and colonization patterns [148].

The relative abundance of functional categories of genes in a microbial ecosystem is an alternative parameter of biodiversity, which can be related to the concept of evenness if one assumes that phylogenetic distance is correlated with functional distance [149]. Note that it is not necessary to make this assumption when analyzing shotgun metagenomes because the relative abundance of different categories of genes can be directly measured. When the phylogenetic and functional measures of biodiversity are compared, very complex interplays between stability and environmental functioning can be revealed, providing the starting material to evaluate and test hypotheses about the ecological role of uncultured genomes obtained from metagenomes. An interesting example of the potential of metagenomics to simultaneously discover new species and provide a broad description of their ecology and natural history is provided in a recent study by Lynch et al. [150]. The authors characterized an uncultured genome sequence obtained from metagenomic data of a volcanic deposit collected 6 km above the sea level in the Atacama Desert. Their study suggested that this uncultured bacterium was indigenous to this harsh environment, with a chemoautotrophic metabolism dependent on trace atmospheric gases [150].

The ecological concept of ecosystem stability is related to biodiversity, and it can be interpreted and measured in different ways [151]. For example, Wittebolle et al. [152] measured the relationship between evenness and stability in different microcosm experiments with denitrifying bacteria. In their study the microcosms were subject to temperature and salt stress, and the stability of the microbial ecosystem was measured as the maintenance of the nitrifying function under stress. The authors showed that the effect of stress on functional stability differed depending on the kind of stress, and that microbial communities with an even functional profile tended to be more resilient to salt induced stress than functionally uneven communities [152]. In the human microbiome, which has become one of the best studied microbial ecosystems, widely different taxonomic compositions have been observed to lead to very similar functional profiles across individuals [153]. This observation of a functional stability supports the insurance hypothesis, being driven by the potential of phylogenetically divergent gut bacteria to acquire similar functions [154, 155]. The relationship between stability and biodiversity is an open research field in microbial community ecology. Top-down metagenomics is providing the means to study this relationship across many different microbial ecosystems, particularly through studies that analyze fluctuations of the taxonomic and functional profiles of communities in space and time [156–159].

## Integrating uncultured genome sequences into a systems biology modeling platform

While the top-down statistical approaches described above provide fundamental information to understand the distribution and ecology of uncultured microorganisms and viruses, they are limited to providing broad-scale predictions that are not always mechanistic. The predictive power of such statistical models can be improved by including more omics data from an environment, such as gene expression, proteomics, and metabolite concentrations [156, 160, 161]. Furthermore, incorporating time series datasets or environmental data such as physicochemical parameters can also contribute to more mechanistic and predictive models [162]. However, a deeper understanding of the biology of new uncultured genomes would come from mechanistic descriptions of the dynamics and biochemical interactions of each subpopulation [163, 164]. Such bottom-up approaches employ computational models to identify robustly predicted patterns in an ecosystem that can subsequently be studied ex silico, for example by exploiting metagenomic datasets. Progress in building genome-scale models for small microbial consortia is beginning to provide a roadmap for describing microbial communities in terms of their individual sub-populations. Below we will discuss several approaches for integrating uncultured genome sequences into computational models, towards describing and understanding the interactions that shape a microbial ecosystem.

### Computational models of microbial cells

The most complete computational model of a cell that integrates several components of the cellular dynamics, such as protein synthesis, and gene expression, was built by Karr et al. [165] for *Mycoplasma genitalium*. This model describes a single organism and reconstructs several patterns of the bacterial cell cycle that are consistent with measurements in vitro [165]. Whole cell models with such level of detail are not currently feasible for most microbes because the roles of novel genes, poorly characterized proteins, and kinetic enzyme parameters remain unknown. Nevertheless, draft biochemical models that propagate and integrate knowledge from known genes that are characterized in other organisms already show significant potential to predict and explain patterns observed in experimental systems [166, 167].

Several different modeling approaches exist that build mechanistic metabolic models of a microbial cell by starting from the genomic sequences, but are beyond the scope of this review [168]. Here we will only point to some of the general principles and possible directions to build predictive models of uncultured genome sequences, and address their role in the community. Our goal is to highlight directions that will position these newly discovered genomes on in silico experimentation platforms. This will accelerate the characterization of these organisms by providing the means to quantitatively describe their interactions with other microbes and the environment, and guide experimental follow-up by providing testable hypotheses about species interactions and their responses to environmental changes.

### Models based on individual genome sequences

When uncultured genome sequences are recovered from an environment by using e.g. metagenomics or single-cell sequencing, the component of their genes that can be annotated can be integrated into a basic biochemical model of directional interactions between proteins and metabolites (for a review of these steps see Refs. [167, 169]). If we assume that several of these models can be inferred for microbes that co-occur within an environment, an important feature that describes their interaction are the exchange reactions that reflect the flow of metabolites in and out of cells. Moreover, the rate by which the cells synthesize biomass components, and the flow of byproducts and secondary metabolites that leave the cell can also be captured. Such metabolic flow models might be used to make predictions about which species grows faster in a given environment [170], the secretion of a products of interest under given conditions [171], the expected biochemical effect of adding or removing a species or metabolite [172], as well as the conditions of the external environment that are required for (mutual) growth [173].

### The flow of metabolites

While some of the information about the metabolic flows can be assessed from the biochemical networks, these networks do not contain information about the kinetic rates of uptake, secretion, and the flow of the metabolites, nor do they contain information about the rates of biomass conversion. In practice, and especially for novel species that contain many unknown genes, we can only reconstruct partial blueprints of the biochemical networks [174]. This suggests that the real flow of metabolites between the organisms consists of complex functions that integrate protein concentrations and affinities, resulting in different reaction rates [175]. Another challenge is capturing the simultaneous reactions from many different biochemical networks within a single model that could contain multiple solutions. Thus, comprehensive models of microbial communities based on individual metabolic networks are not yet available.

### Tackling the complexity of microbial communities

**Fig. 3:**
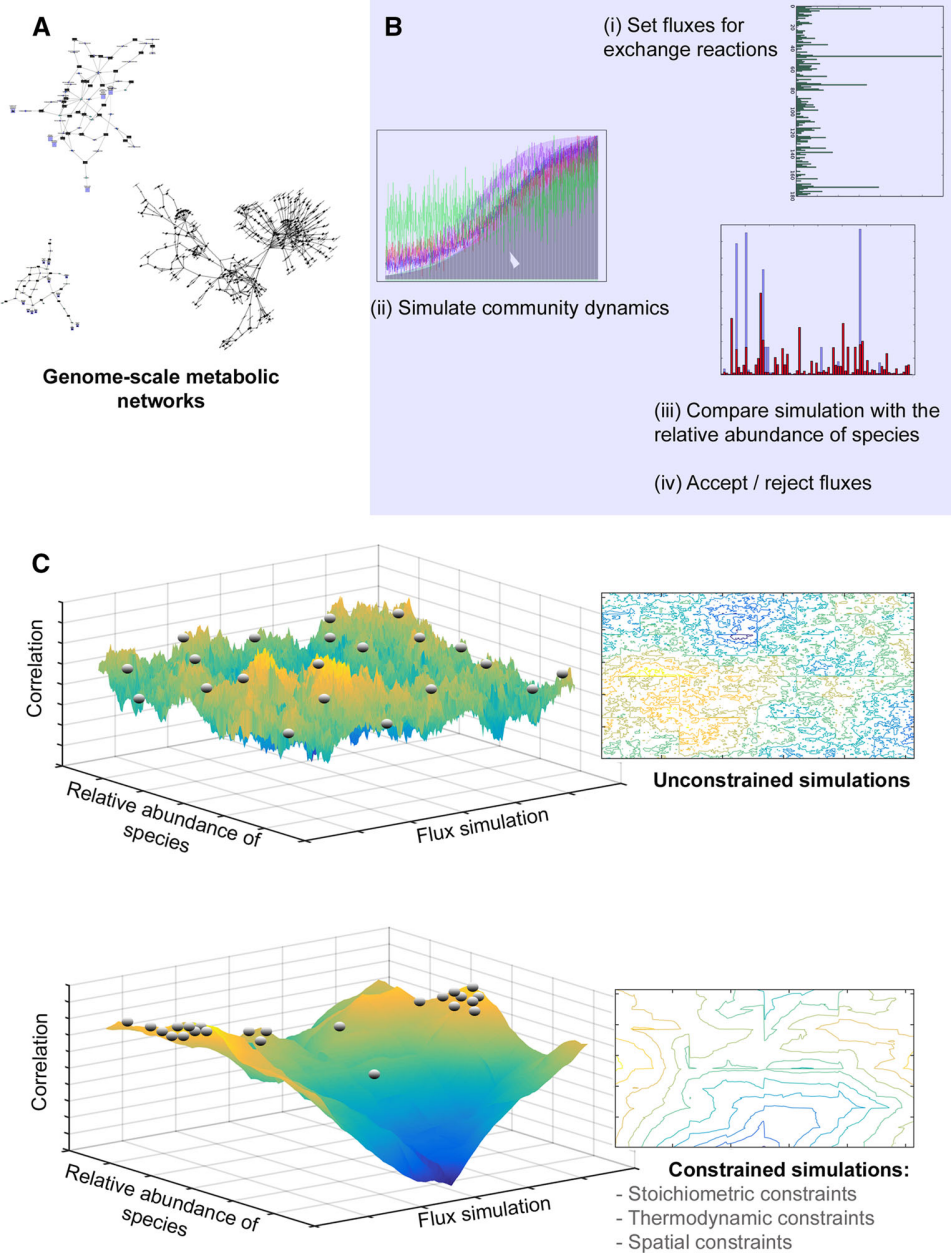
Theoretical representation of the guidelines to build genome-guided simulation-based models for microbial communities applied to a simple model. **a** The model was built for a hypothetical community of biochemical networks corresponding to uncultured genomes. **b** In this model, the variable of interest is the flow-rate of metabolites through exchange reactions in steady-state conformations. Random initial flow-rates were chosen and the growth of the community in a media containing this concentration of metabolites is simulated as in [178]. After equilibrium is reached, the relative abundance of each species is compared to the actual relative abundance from the metagenomic data-set. New values for exchange flow-rates are chosen and simulated, and accepted or rejected according to a stochastic rule or if the predicted relative distribution of species is closer to its actual value. **c** Simulations with or without constraints significantly reduce the solution landscapes indicated by the contour *plots*. The correlations are also significantly higher and have a small number of high-correlation solutions, which can be further studied individually

Small scale models of interacting consortia of few microbes are paving the way for applications to larger communities [171–173, 176–178]. Three important general principles may be extracted from these studies and applied to larger-scale models (Fig. 3). First, the multi-dimensional attractor landscape should be constrained to reduce the degrees of freedom of the solution-space. Second, optimization approaches should be applied to deal with multiple solutions. Third, computational simulations should be used instead of analytical approaches to sample from the possible solution space of multi-level models.

As explained above, the functional insurance hypothesis suggests that there are different possible solutions to how microbial communities may fulfill an environmental niche. In terms of modeling the microbial ecosystem, this can be thought of as different domains of attraction of a highly-dimensional system. This system is subject to important constraints that need to be incorporated in the model. For example, there are hard constraints like the stoichiometric balance of chemical reactions between the metabolites and the second law of thermodynamics, but there are also softer constraints like the spatial boundaries of the system and the diffusion of metabolites that may be captured by stochastic models. Integrating these constraints into systems biological models of the microbial ecosystem can significantly reduce the degrees of freedom of the system, therewith constraining the landscape of its domains of attraction (Fig. 3c). A further way to constrain these models would be to use additional omics datasets to assess gene expression and/or metabolite concentrations [179, 180]. However, even with a constrained landscape of solutions, models of interacting microorganisms could potentially hold an infinite number of solutions. To deal with this degeneracy of solutions, a heuristic approach can be applied that identifies local optima within the attractor landscape that represent biologically meaningful solutions [181, 182].

### Objective functions

Different biological objectives can be defined and expressed as functions in a system of equations with a goal to maximize or minimize this objective, including the objectives that are used in single-species systems [183]. Moreover, approaches to model multiple objectives within a single model have also been explored [184]. The mathematical formulation of a reasonable objective function allows for the optimization of the system for this objective, and depending of the relation of this objective with other variables, the optimization may limit the values and states that may be assumed by the other variables in the system [183]. For example, in a genome-scale model of three gut bacteria, Shoaie et al. [185] used as an objective function the minimization of the uptake of nutrients while maintaining fixed concentrations of biomass. By setting up this configuration, they accurately predicted the concentration of butyrate, CO_2_, and H_2_ obtained from experimental data of germ-free mice colonized with these bacteria [185].

Optimization of objectives in simple systems, such as single-species models, is a straightforward process that usually involves minimizing or maximizing an objective function, while constrained by systems of linear, mixed integer-linear, or simple nonlinear equations. However, optimizing multiple and potentially different objectives from many interacting species that grow at different rates and consume and secrete metabolites at the same time is a significantly more challenging problem. Some of the studies yielding the most promising results have applied approaches that were based on simulating the system, rather than solving it [178, 186]. In simulation-based approaches, the current state of the system is sampled and transition rules are applied that determine its state in the next time point. The system is updated based on these rules and sampled again; this goes on until the system stabilizes in a pattern or distribution.

### Models of microbial consortia: linking to experiments

Using a simulation-based approach for pairs of species, Chiu et al. [178] coupled metabolic networks to Michaelis–Menten dynamics for exchange reactions of the metabolites across the cell membrane. In small time steps, each species would take up, and secrete metabolites proportionally to its biomass and the concentration of the metabolite in the medium. The medium and the biomass of each species were then updated and simulated again, until metabolites were depleted and the growth-rates became zero. This approach predicted the relative abundances of the two bacteria, their temporal growth-rates, and the dynamics of metabolites inside and outside of the cells [178]. A similar approach was used by Harcombe et al. [186], with the addition that they incorporated a spatial lattice into the model where all species could diffuse stochastically. This framework consistently predicted the rate of colony diameter increase in various carbon sources for *E. coli*, as well as the outcome of co-culture experiments of two and three species. Interestingly, an unexpected emergent behavior of the in silico model was confirmed experimentally, showing that the species with the lower growth-rate dominates the co-culture in the long run.

### Linking uncultured viruses to their cellular hosts

Viruses necessarily depend on a cellular host organism for replication, and these virus-host associations can be very specific. Until recently, virus discovery involved isolation of the virus, e.g. by using cell culture or plaque assays, leading to a clear link between a virus and its host. However, with the advent of metagenomic approaches to identify the uncultured viral genome sequences, as described above, virus discovery is no longer dependent on culturing. New bioinformatic approaches are being explored to link viruses to their hosts, based on the information contained in their uncultured genome sequences (Edwards et al., submitted). Signals for virus-host association that have been used in recent studies include the co-occurrence profiles across samples, as described above [63, 107, 187]. Moreover, homology between virus and host genes can indicate a recent gene exchange between their genome sequences, possibly during a recent infection event, and thus homology has also been used to identify virus hosts [63, 188]. For bacteria and archaea, CRISPR spacers that are identified within their genomes can be used to identify the phages that infect them [187, 189], because short fragments from the phage genome sequence are incorporated into CRISPR arrays of the host. Finally, oligonucleotide usage profiles also contain a signal that can be exploited to link an uncultured virus to its cellular host. This depends on viruses ameliorating their genomic oligonucleotide usage to that of the host they infect, for example to avoid recognition by host restriction enzymes, or to adjust their codon usage to match the availability of host tRNAs [190, 191].

Linking uncultured viral genome sequences to a cellular host organism, cultured or uncultured, is an important step towards understanding the microbial ecosystem. Phage-bacterial infection networks (PBIN) describe which phages infect which bacterial hosts [192]. A recent meta-analysis of PBIN showed a characteristic structuring that is globally modular and locally nested [193, 194]. This means that bacteria and phages from different locations are mostly incompatible (global modularity). Within one location, phages co-exist with varying host specificity (local nestedness), e.g. generalist phages that infect many bacteria, and specialist phages that infect only one bacterium. Phage predation can have a huge impact on microbial ecology, maintaining biodiversity through Kill-the-Winner dynamics [195], and releasing nutrients through the viral shunt [196]. Incorporating phage predation into ecosystem models will allow the effects of this important parameter in microbial ecology to be studied [196, 197].

## Conclusions

Obtaining the genome sequences of uncultured microbes and viruses in metagenomes is one of the most promising areas of research in microbiology. Novel strategies to sample and sequence environmental metagenomes as well as significant advances in bioinformatics and data recycling are increasing our knowledge of uncultured microorganisms. With metagenomic approaches, we can discover the identity, evolution, gene composition, distribution, and ecological patterns of uncultured microbes and viruses. Our challenge now is to integrate this knowledge into predictive analytical models of microbial ecosystems that incorporate the knowledge that can be mined from both uncultured and cultured genome sequences [163]. It is still difficult to realistically capture important properties of microbial ecosystems in analytical models, such as spatial structuring, diffusion of nutrients, energy barriers, selective sweeps by bacteriophages, and the immune system in case of host-associated microbiota. Recent progress has shown that the way forward is to apply modeling through multi-step simulation-based approaches. Although there are still many caveats to these approaches, we believe that future development in this area will provide outstanding tools to mechanistically understand the biology of uncultured microbes. Some of the variables that could be predicted by these models and experimentally validated are energy flux patterns, cross feeding patterns, and the dynamics of diversity within the community of study. If a community is described in terms of energy and matter flow, it can also be compared in these terms, providing not only a unique insight into the evolutionary processes that have shaped microbial communities, but also informing in a precise and mechanistic manner how these balances could be changed, or how changes in these balances impact biodiversity. Systems biology platforms with these potentials are the immediate goals for further advances in discovering and understanding the microscopic and submicroscopic biosphere. The major remaining challenges include providing the expanding number of sequences available with reliable annotations, and incorporating these into consistent models of interacting microbes and viruses in the natural ecosystem. To conclude, the exciting field of uncultured microbe and virus discovery, and the study of interactions in natural microbial ecosystems has grown with metagenomics throughout the past decade, and recent developments hold promise of many more discoveries in the near future.
